# Genetic Rearrangements of Six Wheat–*Agropyron cristatum* 6P Addition Lines Revealed by Molecular Markers

**DOI:** 10.1371/journal.pone.0091066

**Published:** 2014-03-04

**Authors:** Haiming Han, Li Bai, Junji Su, Jinpeng Zhang, Liqiang Song, Ainong Gao, Xinming Yang, Xiuquan Li, Weihua Liu, Lihui Li

**Affiliations:** National Key Facility for Crop Gene Resources and Genetic Improvement (NKCRI), Institute of Crop Sciences, Chinese Academy of Agricultural Sciences, Beijing, China; Kansas State University, United States of America

## Abstract

*Agropyron cristatum* (L.) Gaertn. (2*n* = 4*x* = 28, **PPPP**) not only is cultivated as pasture fodder but also could provide many desirable genes for wheat improvement. It is critical to obtain common wheat–*A. cristatum* alien disomic addition lines to locate the desired genes on the P genome chromosomes. Comparative analysis of the homoeologous relationships between the **P** genome chromosome and wheat genome chromosomes is a key step in transferring different desirable genes into common wheat and producing the desired alien translocation line while compensating for the loss of wheat chromatin. In this study, six common wheat–*A. cristatum* disomic addition lines were produced and analyzed by phenotypic examination, genomic *in situ* hybridization (GISH), SSR markers from the **ABD** genomes and STS markers from the **P** genome. Comparative maps, six in total, were generated and demonstrated that all six addition lines belonged to homoeologous group 6. However, chromosome 6**P** had undergone obvious rearrangements in different addition lines compared with the wheat chromosome, indicating that to obtain a genetic compensating alien translocation line, one should recombine alien chromosomal regions with homoeologous wheat chromosomes. Indeed, these addition lines were classified into four types based on the comparative mapping: 6**P_I_**, 6**P_II_**, 6**P_III_**, and 6**P_IV_**. The different types of chromosome 6**P** possessed different desirable genes. For example, the 6**P_I_** type, containing three addition lines, carried genes conferring high numbers of kernels per spike and resistance to powdery mildew, important traits for wheat improvement. These results may prove valuable for promoting the development of conventional chromosome engineering techniques toward molecular chromosome engineering.

## Introduction

Transferring desirable genes from wild relatives into common wheat is an important strategy for wheat breeding. Since McFadden [1] first transferred a stem rust resistance gene from *Triticum dicoccum* into common wheat, breeders and geneticists have transferred numerous useful genes into wheat through genetic manipulation. The range of hybridizations between wheat and its wild relatives has been extended continuously. All the genera of the tribe Triticeae have been successfully hybridized with wheat, including 23 genomes and more than 100 alien genes that have been transferred into wheat and named [2], [3]. However, due to insufficient compensation or genetic drag, the alien genes with important roles in wheat breeding involve only 5 linkage groups, including the following 15 genes: *Lr24/Sr24*, *Sr26* from *Thinopyrum elongatum*, *Sr36/Pm6* from *Triticum timopheevii*, *Lr26/Sr31/Yr9/Pm8* from the translocation line T1BL·1R#1S, *Gb2/Pm17* from T1AL·1R#2S of *Secale cereale*, and *Yr17/Lr37/Sr38/Cre5* from *Aegilops ventricosa* [4], [5], [6].

Producing wheat–alien chromosome disomic addition lines and analyzing their genetic constitutions is a key step for the effective transfer of useful genes. Since the 1950s, many wheat–alien chromosome disomic addition lines have been produced, such as rye [7], barley [8], *Dasypyrum villosum* [9], and several species of the genera *Agropyron* [25], [26], *Aegilops* [10], and *Thinopyrum* [11]. These addition lines are often used as bridge materials to transfer desirable genes to wheat. Understanding the genetic constitutions of addition lines could be helpful for producing compensating translocations for the transfer of genes from alien chromosomes into the wheat genomes. Disomic addition lines can be identified by means such as morphological analysis, chromosome banding, *in situ* hybridization, and molecular markers. Molecular markers have been widely used to determine the homoeologous relationships between alien and wheat chromosomes [27], [62], [63], [64], [65].

*Agropyron* Gaertn. (**P** genome), a perennial genus of the tribe Triticeae, not only is cultivated as pasture fodder but also could provide many desirable genes for wheat improvement [12], [13], [14]. Previous wide crosses between common wheat and *Agropyron* have been considered unsuccessful [15], [16]. It may not be possible to transfer genes from *Agropyron* to *Triticum* even if the intergeneric hybrids can be obtained [12]. However, the application of embryo rescue has enabled the successful hybridization of common wheat with *Agropyron* species, such as *A. cristatum*, *A. desertorum*, *A. fragile* and *A. michnoi* [17], [18], [19], [20], [21], [22], [23], [24]. In our laboratory, the common wheat Fukuhokomugi was used as the maternal parent in a hybridization with the *A. cristatum* accession Z559, which originated from Xinjiang, China [23]. Following several generations of backcrossing or selfing, a series of disomic addition lines was obtained [25], [26], including the stable disomic addition line 4844-12, which possessed large spikes with multiple florets and grains and was identified as a 6**P** disomic addition line by genetic control analysis [27]. The successful distant hybridization between common wheat and *A. cristatum*, as well as the development of addition lines, are the foundation of the transfer of desirable genes from *A. cristatum* to wheat.

In this study, six wheat–*A. cristatum* disomic addition lines were identified using GISH, SSR and STS molecular markers, and their genetic constitutions and morphology were comparatively analyzed. The aim of this work was to provide guidance for transferring desirable compensating genes and for improving the utilization efficiency of wheat breeding through distant hybridization.

## Materials and Methods

Our experiment was carried out at the CAAS Experiment Station, and the studies did not involve the protected area of land and endangered or protected species.

### Plant Materials

Six wheat–*A. cristatum* alien addition lines were obtained after several generations of backcrossing or selfing following the hybridization of the common wheat Fukuhokomugi with *A. cristatum* accession Z559 (2*n* = 4*x* = 28, **PPPP**). The accession numbers of these lines are 4844-12, 5113, 5114, 5106, II-26, and II-29-2i (Table 1), and one of them (4844-12) was 6**P** disomic addition line identified previously by Wu et al. [27].

**Table 1:** Six addition lines from *Triticum aestivum* and *A. cristatum.*

Accession no.	Original F_1_ hybrids	Generations
4844-12	FC-1	F_6_
5113	FC-1	BC_1_F_2_
5114	FC-1	BC_1_F_2_
5106	FC-1	BC_1_F_2_
II-26	FC-2	BC_3_F_1_
II-29-2i	FC-2	BC_3_F_1_

### Morphology of Addition Lines

During the 2010–2011 wheat growing season, these lines were planted in a field trial with two replicates at the CAAS Experiment Station in Beijing, China. Each line was planted in three 2-m rows spaced 30 cm apart, with 30 seeds in each row. For each replicate, ten plants were harvested to evaluate their agronomic traits, including grain number per spikelet, grain number per spike, and thousand-grain weight, etc. The means for the different lines were compared using Fisher’s LSD (*P*<0.01) in the SAS package (V8.1, SAS Institute Inc., Cary, NC, USA). The evaluation of powdery mildew resistance was performed under field conditions by inoculating the adult plants with *Erysiphe graminis* f. sp. *tritici* (Egt) isolate E09. The scale described by Li et al. [28] was used to score the infection types as HR (highly resistant), R (resistant), MR (moderately resistant), S (susceptible), or HS (highly susceptible).

### Chromosome Preparation and GISH

To determine whether there were *A. cristatum* chromosomes in each line, the chromosome composition was confirmed using GISH analysis. The methods for preparing chromosomes from the plant root tip cells and pollen mother cells (PMCs) were described in Cuadrado et al. [29]. The cytological observations were performed using a BX51 Olympus phase-contrast microscope (Olympus Corp., Tokyo, Japan).

*A. cristatum* genomic DNA was used as a probe to detect the **P** genome chromosome, and Fukuhokomugi genomic DNA was used for blocking. GISH was performed on the root tip cells and PMCs in accordance with the basic method described by Cuadrado et al. [29] and the improved procedure described by Liu et al. [30]. The in situ hybridization images were obtained using an Olympus AX80 (Japan) fluorescence microscope and were processed using Photoshop CS 3.0 (Adobe, San Jose, CA, USA).

### Molecular Marker Analysis

The SDS method was used to extract genomic DNA from *A. cristatum* (Z559), the recipient parent Fukuhokomugi and the plants that had been identified as disomic addition lines [31].

In total, 904 SSR primer pairs and 422 EST-SSR primer pairs [32], [33], [34], [35], [36] were used to analyze the homoeologous relationships between the **P** genome chromosomes and the wheat genome chromosomes. In total, 2,815 sequence-tagged site (STS) markers were designed based on *A. cristatum* expressed sequence tags (ESTs) and suppression subtractive hybridization between 4844-12 and the recipient parent, Fukuhokomugi. These STS markers were used to analyze the genetic constitutions of the six addition lines. PCR was performed as previously described by Luan et al. [37]. The amplification products were separated on 6% denaturing polyacrylamide gels and were visualized by silver staining.

A cluster analysis of the six addition lines, based on the STS markers, was performed to generate a dendrogram using the unweighted pair group method with arithmetic averages (UPGMA) in the SAS software package (V8.1, SAS Institute Inc., Cary, NC, USA).

### Construction of Chromosome 6P Comparative Maps

Comparative maps of chromosome 6**P** were constructed based on the relative locations of the STS markers specific to the 6**P** disomic addition lines. The relative locations of the 6**P**-specific markers were determined by comparing the ESTs corresponding to the markers with the deletion-mapped ESTs from hexaploid wheat using local BLASTN and TBLASTX algorithms. The sequence comparison was performed as previously described [38], [39]. The high-scoring pairs (HSPs) with an E-value greater than 1E-5 were rejected. ESTs with matched wheat ESTs that were not mapped or that mapped only to a chromosome or chromosome arm without clear loci, were excluded from the analysis [40], [41]. The chromosomal breakpoints were determined by deletion mapping [42], [43]. Homoeologous group 6 of the **A**, **B**, and **D** wheat genomes was combined into a single consensus wheat genome to construct the group 6 consensus chromosome bin map, as previously described [44]. The breakpoints on this consensus chromosome bin map divided the chromosome into a larger number of bins than the individual chromosomes. By comparison, the specific markers that contained location information were used to construct the comparative maps. Details of the mapped wheat ESTs can be found at http://wheat.pw.usda.gov/cgi-bin/westsql/map_locus.cgi.

## Results

### GISH Detection of the Six Addition Lines

**Figure 1:**
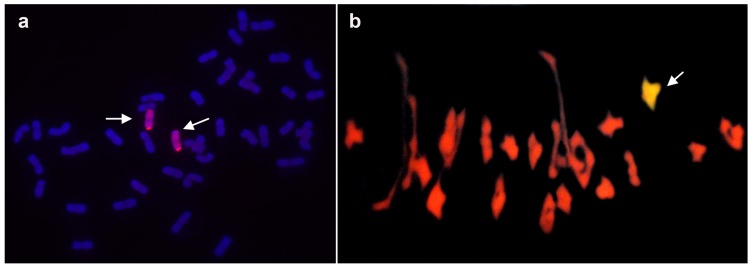
GISH patterns of wheat-*A. cristatum* addition line II-26. (a) Chromosomes in red are *A. cristatum* chromosomes. The arrows indicate two alien P chromosomes in the addition line at mitotic metaphase. (b) Chromosomes in yellow-green are *A. cristatum* chromosomes. The arrow indicates a ring bivalent formed between the two P chromosomes at meiotic metaphase I.

GISH was performed to detect *A. cristatum* chromosomes in the six addition lines 4844-12, 5113, 5114, 5106, II-26, and II-29-2i. Mitotic GISH revealed that the root-tip cells contained two *A. cristatum* chromosomes of the addition line II-26 (Figure 1a). Meiotic GISH indicated that two **P** genome chromosomes were paired in the PMCs at meiotic metaphase I of the addition line II-26 (Figure 1b). Observations from the other addition lines were the same with that from the addition line II-26 (data not shown), which confirmed that all the materials were wheat–*A. cristatum* disomic addition lines (2*n* = 44).

### Homoeology of *A. cristatum* Chromosomes with Wheat Chromosomes

**Figure 2:**
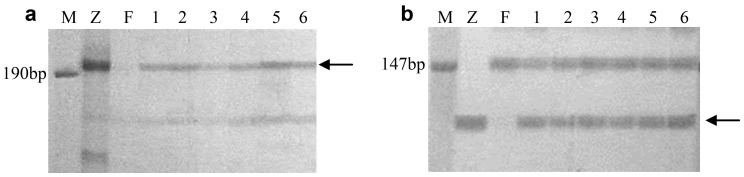
PCR amplification profiles using primers *Xgwm113* (a) and *Cfd80* (b). M: DNA ladder; Z: *A. cristatum* (Z559); F: Fukuhokomugi; 1: II-29-2i; 2∶5113; 3: II-26; 4∶5106; 5∶4844-12; 6∶5114. The arrows indicate the diagnostic bands of P chromatin.

A total of 904 SSR and 422 EST-SSR markers distributed over seven wheat homoeologous groups were used to analyze Z559 and Fukuhokomugi. The marker analysis showed that 334 (37.0%) of the SSR and 229 (54.3%) of the EST-SSR markers were polymorphic between Z559 and Fukuhokomugi (Table 2). Markers specific to *A. cristatum* accounted for 42.5% of the total and were distributed over seven wheat homoeologous groups. The 563 polymorphic SSR and EST-SSR markers were further used to analyze the six wheat–*A. cristatum* disomic addition lines and their parents, Z559 and Fukuhokomugi. Of the polymorphic markers, fifty-one successfully amplified **P** genome-specific bands in the addition lines (Table 3), e.g., *Xgwm113* and *Cfd80* on chromosomes 4**B** and 6**A**/6**D**, respectively (Figure 2). The largest number of specific markers, 20 (39.2%), belonged to homoeologous group 6. Among the other markers, 7 (13.7%) belonged to homoeologous group 1, 8 (15.7%) to group 2, 4 (7.8%) to group 3, 4 (7.8%) to group 4, 4 (7.8%) to group 5, and 4 (7.8%) to group 7.

**Table 2:** Polymorphism of SSR and EST-SSR markers between *A. cristatum* and Fukuhokomugi.

Type of marker	Series	Number of detected markers	Number of polymorphic markers	Proportion (%)
SSR	*Xgwm*	233	76	32.62
	*Xgdm*	69	34	49.28
	*Barc*	206	75	36.41
	*Wmc*	261	104	39.85
	*Cfd*	109	36	33.03
	*Cfa*	26	9	34.62
	Total	904	334	36.95
EST-SSR	*Ksum*	51	20	39.21
	*Cnl*	19	7	36.84
	*Cwem*	26	12	46.15
	*Cfe*	156	73	46.79
	*Swes*	170	117	68.82
	Total	422	299	54.27
Total		1326	563	42.46

**Table 3:** PCR amplification of P chromatin in the six addition lines using SSR and EST-SSR markers.

Primers	Chromosome	Z559	Fukuhokomugi	II-29-2i	5113	II-26	5106	4844-12	5114
*Wmc716*	1AL	+	−	+	+	+	+	+	+
*Xgwm268*	1BL	+	−	−	−	−	−	+	−
*Swes215*	1B	+	−	+	+	+	+	+	+
*Xgwm232*	1DL	+	−	−	−	−	+	−	−
*Cfe26*	1A1B	+	−	−	+	+	+	+	−
*Swes145*	1A1B	+	−	−	−	−	+	−	−
*Swes98*	1B1D	+	−	+	+	+	+	+	+
*Xgwm265*	2AL	+	−	+	+	+	+	+	+
*Xgwm339*	2AL	+	−	+	+	+	+	+	+
*Xgwm425*	2AL	+	−	+	+	+	+	+	+
*Xgwm614*	2AS	+	−	+	−	−	+	+	+
*Xgdm107*	2DS	+	−	−	−	−	+	−	−
*Xgdm35*	2DS	+	−	+	+	+	+	+	+
*Xgwm455*	2DS	+	−	+	+	+	+	+	+
*Barc335*	2D	+	−	+	+	+	+	+	+
*Xgwm674*	3AS	+	−	+	+	+	+	+	+
*Wmc612*	3BS	+	−	−	−	−	−	+	−
*Wmc623*	3BS	+	−	+	+	+	+	+	+
*Xgdm38*	3DL	+	−	+	+	+	+	+	+
*Xgwm113*	4BL	+	−	+	+	+	+	+	+
*Ksum154*	4BL	+	−	−	−	−	+	+	−
*Xgdm61*	4DL	+	−	+	+	+	+	+	+
*Wmc617*	4AL4BL4DS	+	−	+	+	+	+	+	+
*Swes71*	5B	+	−	−	+	+	−	−	−
*Xgdm116*	5DL	+	−	−	−	−	−	+	−
*Xgwm654*	5DL	+	−	+	+	+	+	+	+
*Cnl142*	5BS	+	−	+	+	+	+	+	+
*Cfe132*	6A	+	−	+	+	+	+	+	+
*Cfe179*	6A	+	−	+	+	+	+	+	+
*Xgdm147*	6B	+	−	−	+	+	−	−	−
*Cfe32*	6B	+	−	+	+	+	+	+	+
*Cnl64*	6BS	+	−	+	+	+	+	+	+
*Cnl113*	6BS	+	−	+	+	+	+	+	+
*Swes180*	6BS	+	−	+	+	+	+	+	+
*Barc79*	6BL	+	−	+	+	+	+	+	+
*Xgwm608*	6BL	+	−	+	+	+	+	+	+
*Xgwm644*	6BL	+	−	+	+	+	+	+	+
*Xgdm61*	6BL	+	−	−	+	+	−	−	−
*Xgwm311*	6BL2A	+	−	+	+	+	+	+	+
*Barc123*	6DS	+	−	+	+	+	+	+	+
*Xgwm469*	6DS	+	−	+	+	+	+	+	+
*Barc21*	6DL	+	−	+	+	+	+	+	+
*Barc175*	6D	+	−	+	+	+	+	+	+
*Barc146*	6A6B	+	−	+	+	+	+	+	+
*Cfd80*	6A6D	+	−	+	+	+	+	+	+
*Swes2*	6A6BL6DS	+	−	−	−	−	−	+	−
*Cfe2*	6A6B6D	+	−	−	−	−	−	+	−
*Barc1167*	7A	+	−	+	+	+	+	+	+
*Xgwm282*	7AL	+	−	+	+	+	+	+	+
*Wmc76*	7BL	+	−	−	−	−	+	−	−
*Swes157*	7A7D	+	−	−	−	−	+	−	−

+, − represent positive and negative amplification of SSR and EST-SSR markers, respectively.

**Figure 3:**
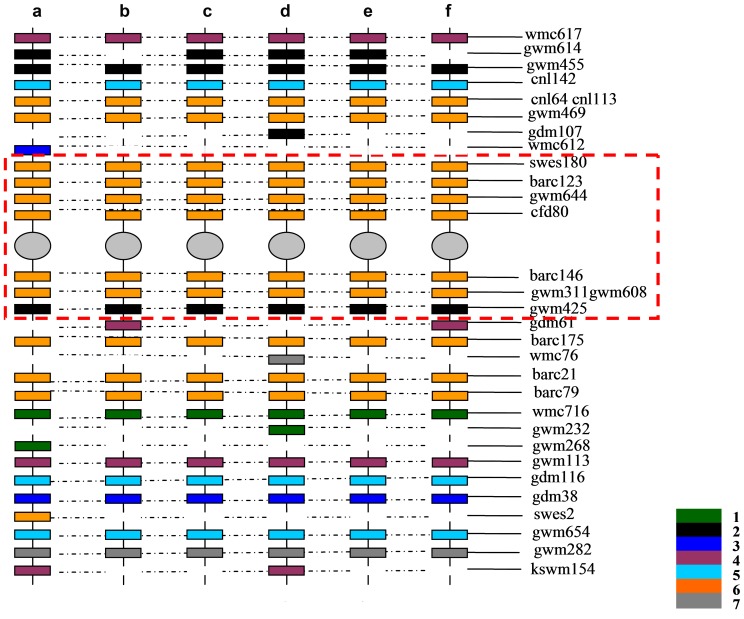
A map of the *A. cristatum* chromosomes in the six addition lines. a, b, c, d, e, and f represent the *A. cristatum* chromosomes in the disomic addition lines 4844-12, 5113, 5114, 5106, II-29-2i, and II-26, respectively. The colored strips and the numbers 1, 2, 3, 4, 5, 6, and 7 represent the homoeologous groups 1 to 7 in wheat. The markers in the dotted box indicate the same loci in the pericentromeric region of the six addition lines.

According to the genetic maps of SSR and EST-SSR [32], [33], [34], [35], [36], 32 markers which amplified specific bands in the wheat–*A. cristatum* addition lines were located on 21 pairs of wheat chromosomes. A map of chromosome 6**P** was generated based on the locations of the above markers on wheat chromosomes **A**, **B**, and **D** (Figure 3). This map indicated that 21.9% of the SSR and EST-SSR markers specific to the **P** genome were located near the pericentromeric region of 6**A**, 6**B**, and 6**D**; the remaining specific markers were distributed in the distal region of the chromosome (Figure 3). The homoeologous group of one chromosome could be determined by the markers clustered in the pericentromeric region, for the sequence of this region was highly conserved. Therefore, the *A. cristatum* chromosomes in the six addition lines (4844-12, 5113, 5114, 5106, II-26, and II-29-2i) were homoeologous to group 6 of common wheat and were thus designated as 6**P**. The mapped molecular markers specific to addition lines were primarily related to homoeologous group 6 (43.8%); the remaining markers (total 56.2%) were related to other homoeologous groups. This finding indicates that chromosome 6**P** may have undergone rearrangements.

### Construction of Chromosome 6P Comparative Maps and Further Confirmation of Rearrangements

**Figure 4:**
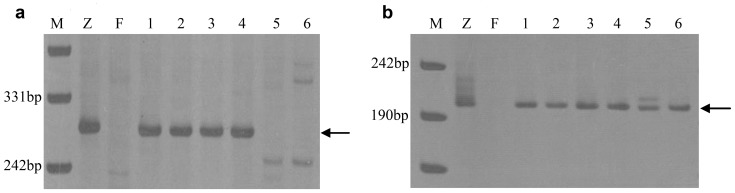
PCR amplification profiles using STS markers *Agc70446* (a) and *Agc13186* (b). M: DNA ladder; Z: *A. cristatum* (Z559); F: Fukuhokomugi; 1∶4844-12; 2∶5113; 3∶5114; 4∶5106; 5: II-26; 6: II-29-2i. The arrows indicate diagnostic bands of P chromatin.

**Figure 5:**
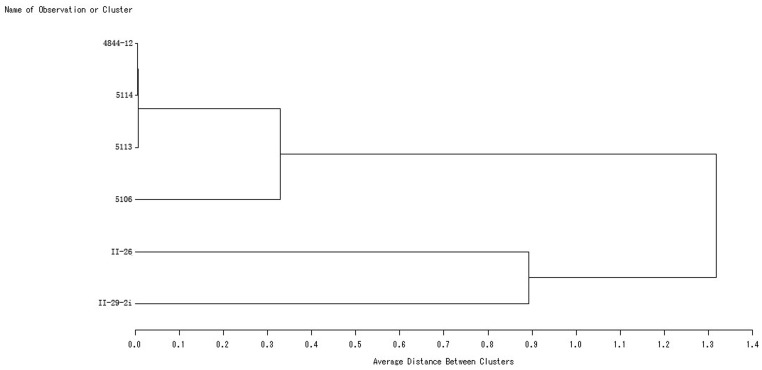
Cluster tree of the six addition lines based on STS markers and generated using UPGMA.

2,815 STS markers were used to analyze *A. cristatum* accession Z559, common wheat Fukuhokomugi and the six wheat–*A. cristatum* 6**P** disomic addition lines (4844-12, 5113, 5114, 5106, II-29-2i, and II-26). The results demonstrated that 688 primer pairs amplified **P** genome-specific bands, and the amplification results differed among the addition lines. There were 14 types of results (Table 4). Type 1 (Figure 4a) were the largest group (38.5%), followed by the II-29-2i-specific markers (27.2%). Although 44 markers were common to all six addition lines (Figure 4b), these represented only 6.4% of the total, and even though there were many common specific markers for 4844-12, 5113, and 5114, the minor differences could also be found by molecular marker analysis. This result indicated that the six addition lines differed in terms of chromosome 6**P** and genetic constitution. The cluster analysis performed using UPGMA (SAS V8.1 software) indicated that the six addition lines could be divided into four groups (Figure 5): 6**P_I_** (4844-12/5113/5114), 6**P_II_** (5106), 6**P_III_** (II-29-2i), and 6**P_IV_** (II-26).

**Table 4:** Types of STS markers specific to chromosome 6P in wheat–*A. cristatum* addition lines.

Type	Z559	Fukuhokomugi	4844-12	5113	5114	5106	II-26	II-29-2i	Number of markers	Percentage (%)
1	+	−	+	+	+	+	−	−	265	38.52
2	+	−	+	+	+	+	+	+	44	6.40
3	+	−	+	+	+	+	+	−	26	3.78
4	+	−	+	+	+	+	−	+	15	2.18
5	+	−	−	−	−	−	−	+	187	27.18
6	+	−	−	−	−	−	+	−	29	4.22
7	+	−	−	−	−	+	+	−	69	10.03
8	+	−	−	−	−	+	−	+	23	3.34
9	+	−	−	−	−	+	+	+	20	2.91
10	+	−	−	−	−	+	−	−	6	0.87
11	+	−	+	−	+	−	+	+	1	0.15
12	+	−	+	−	+	+	−	−	1	0.15
13	+	−	+	−	+	−	−	−	1	0.15
14	+	−	−	+	+	+	−	+	1	0.15
Total	688	0	353	351	354	470	189	290	688	100

+, − represent positive and negative amplification of STS markers, respectively.

A large number of the ESTs that were physically mapped in wheat chromosome bins provided valuable information about the chromosome or genome constitution. A sequence comparison was performed between 688 corresponding ESTs of the STS markers specific to the wheat–*A. cristatum* addition lines and wheat ESTs with known physical locations [42], [43]. 160 ESTs of *A. cristatum* could match mapped wheat ESTs by this comparison. The locations of the 6**P**-specific markers were determined based on the information derived from the matched wheat ESTs.

**Figure 6:**
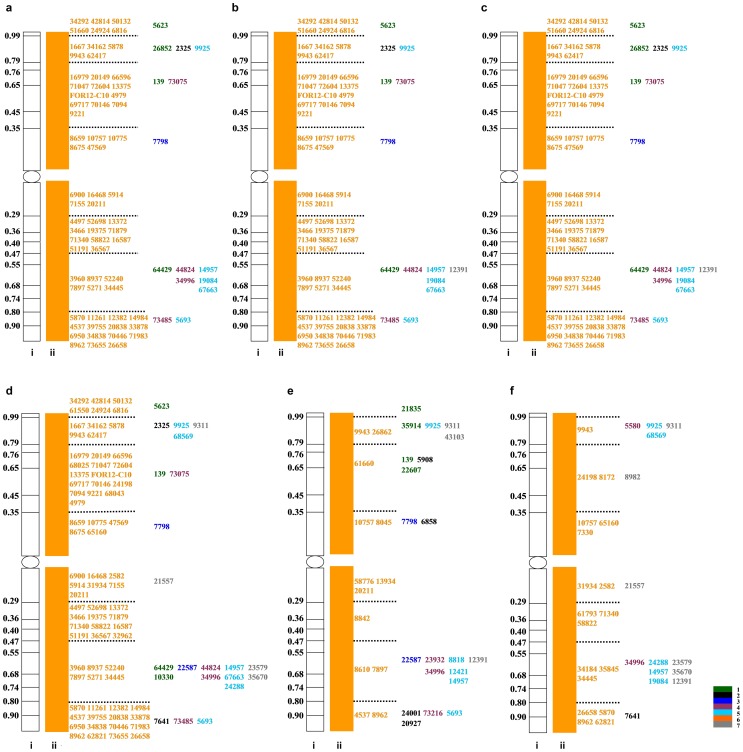
Comparative maps of *A. cristatum* chromosome 6P in the six addition lines. a, b, c, d, e, and f represent the *A. cristatum* chromosomes in the disomic addition lines 4844-12, 5113, 5114, 5106, II-29-2i, and II-26, respectively. i: Consensus bin map of wheat group 6; ii: The specific markers distributed in different bins along the chromosome 6P. The colored strips and the numbers 1, 2, 3, 4, 5, 6, and 7 represent the homoeologous groups 1 to 7. The colored markers which belonged to different homoeologous groups were consistent with the colored strips. The ‘*Agc*’ of the marker names were ellipsis in the maps.

Using the wheat homoeologous group 6 consensus chromosome bin map as a reference, chromosome 6**P** was divided into 8 bins (6S-0.00–0.35, 6S-0.35–0.79, 6S-0.79–0.99, and 6S-0.99–1.00; 6L-0.00–0.29, 6L-0.29–0.47, 6L-0.47–0.80, and 6L-0.80–1.00) based on the number and distribution pattern of the 6**P**-specific STS markers. The STS markers that mapped to a portion of a chromosome arm containing more than one bin were excluded from the analysis. Thus, 124 of 160 markers were mapped to different chromosome 6**P** bins, and six different comparative maps were constructed (Figure 6). There were 80, 79, 81, 94, 34, and 32 markers along the six 6**P** chromosomes. It was found on the maps that most of the STS markers (81.3%, 82.3%, 80.3%, 76.6%, 38.2%, and 56.3%) belonged to homoeologous group 6, and the remaining markers which distributed mainly in distal regions (18.8%, 17.7%, 19.8%, 23.4%, 61.8%, and 43.8%) belonged to other homoeologous groups. Therefore, these results are consistent with those from the wheat molecular markers and further confirmed that the *A. cristatum* chromosomes had a homoeologous relationship with group 6 of wheat.

The six addition lines were divided into four types (6**P_I_**, 6**P_II_**, 6**P_III_**, and 6**P_IV_**) based on a cluster analysis. There were large differences among the different chromosome 6**P** types in terms of both the number of markers and the distribution density on chromosome 6**P**. Although all of the added *A. cristatum* chromosomes were chromosome 6**P**, their molecular markers of homoeologous group 6 exhibited varying degrees of difference. The markers in the dotted box of Figure 3 showed the same loci in the pericentromeric region of the six addition lines. However, Figure 6 showed the homoeologous group 6 markers in the pericentromeric region were not completely identical among types 6**P_I_**–6**P_IV_**; moreover, there were several markers belonging to other homoeologous groups. The results further demonstrated that the genetic constitution of chromosome 6**P** differed among the six addition lines. Additionally, the results indicated that different types of chromosome 6**P** underwent obvious structural rearrangements compared with the wheat genome, and these rearrangements occurred mainly in the distal region of chromosome 6**P**.

### Chromosomal Locations of Desirable Alien Genes in Addition Lines

**Figure 7:**
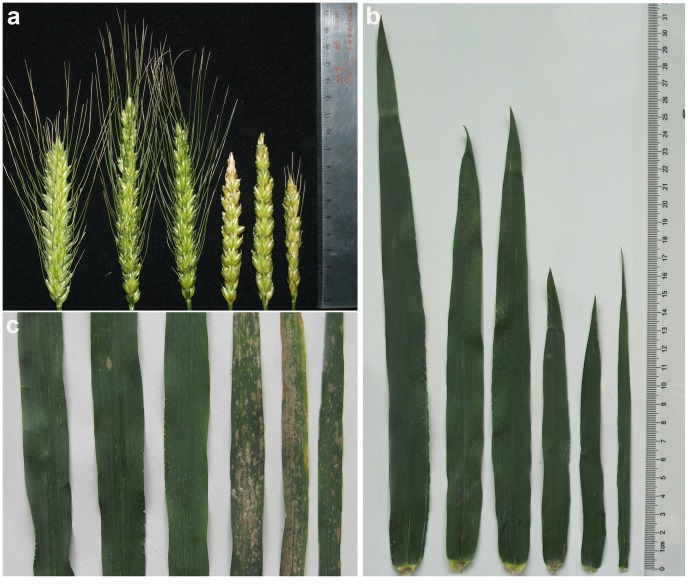
Morphological comparison of the six wheat-*A. cristatum* disomic addition lines. (a) Spike morphology (b) Morphology of flag leaves. (c) Symptoms of powdery mildew resistance. The materials from left to right in a, b, and c are 4844-12, 5113, 5114, 5106, II-26, and II-29-2i, respectively.

The main agronomic traits of the six wheat–*A. cristatum* addition lines are shown in table 5. All of agronomic traits were analyzed by an analysis of variance with repeated measures (ANOVAR, *P<*0.01) [45]. We found significant differences in grain number per spikelet and grain number per spike; 6**P_I_** (comprising three addition lines: 4844-12, 5113, and 5114) was significantly higher than the wheat parent, Fukuhokomugi, with no infertile spikelets (Figure 7a). Previous study has reported that a gene(s) controlling high numbers of kernels per spike is located on chromosome 6**P** of the wheat–*A. cristatum* addition line 4844-12 [27]. A further comparative analysis of the genetic constitutions of 5113, 5114, and 4844-12 revealed that their *A. cristatum* chromosomes were all 6**P_I_**, suggesting that the added chromosome 6**P_I_** of 4844-12, 5113, and 5114 carried a gene(s) conferring high numbers of kernels per spike.

**Table 5:** Comparison of main agronomic traits among the six wheat–*A. cristatum* disomic addition lines.

Addition line	Chromosome composition	Spike length (cm)	Spikelet number per spike	Grain number per spikelet	Grain number per spike	Thousand-grain weight	Powdery mildew resistance
Fukuhokomugi	42W^a^	7.36E^b^	14.80C	4.00C	40.50B	24.60BC	HS
4844-12	42W+2P_I_	10.39BC	22.00A	6.40A	95.70A	43.19A	HR
5113	42W+2P_I_	11.88A	22.30A	5.40B	88.40A	20.64C	HR
5114	42W+2P_I_	10.54B	20.70A	6.30A	87.50A	29.23B	HR
5106	42W+2P_II_	8.83D	17.70B	2.90D	25.20C	37.62A	HS
II-29-2i	42W+2P_III_	7.13E	17.60B	3.10D	32.60BC	28.86B	S
II-26	42W+2P_IV_	9.36CD	18.50B	2.70D	33.60BC	38.11A	HS

a P and W indicate *A. cristatum* and wheat chromosomes, respectively.

b Means followed by the same letter are not significantly different (*P<*0.01) based on Fisher’s LSD.

The evaluation of the six addition lines for resistance to powdery mildew showed that the addition lines differed in their degree of powdery mildew resistance. 4844-12, 5113, and 5114 were highly resistant to Egt isolate E09, whereas II-29-2i was susceptible and II-26 and 5106 were highly susceptible to E09 (Figure 7c). Comparison of the powdery mildew resistance of the recipient wheat Fukuhokomugi (highly susceptible) and the alien donor *A. cristatum* (immune) indicated that 6**P_I_** carried genes conferring not only high numbers of kernels per spike but also resistance to powdery mildew.

In addition, the sizes of the flag leaves in the six addition lines differed; II-29-2i had an especially slender flag leaf (Figure 7b), which was similar to that of *A. cristatum*. Moreover, there were no significant differences between II-29-2i and the wheat parent, Fukuhokomugi, in grain number per spike or thousand-kernel weight (Table 5). This specific trait may be useful in wheat improvement programs.

## Discussion

In contrast to conventional disomic addition lines, this study identified 4 types of wheat–*A. cristatum* 6**P** disomic addition lines based on the alien chromosome (6**P**) rearrangements. Theoretically, the number of a complete set of wheat–*A. cristatum* disomic addition lines should be seven, with one pair of alien chromosomes corresponding to one disomic addition line, and there should be only one 6**P** disomic addition line. In fact, six wheat–*A. cristatum* 6**P** disomic addition lines in this study were obtained. They were derived from two F_1_ hybrids and could be divided into four types by molecular marker analysis, indicating that the two F_1_ hybrids likely contained a different **PP** combination from the tetraploid parent, *Agropyron cristatum*. *Agropyron* are cross-pollinating plants. The tetraploid *A. cristatum* originates from derivatives of hybridizations between diploid *A. cristatum* and *A. mongolicum*. Although the diploid *A. cristatum* and *A. mongolicum* contain the same basic **P** genome, their **P** genomes exhibited rearrangements and variation [46], [47]. The two **P** genomes exhibit segmental autosomy in the tetraploid *A. cristatum* [48] and are distinguished from each other by structural rearrangements [46]. Studies of genetic diversity have shown that there are genetic variations among different individuals within one population [66], [67], thus there might be large genetic differences between the **P** chromosomes of the two F_1_ hybrids. We accordingly speculated that the major rearrangements of **P**-genome chromosomes might have been present in the tetraploid *A. cristatum* before the hybridization of wheat with *A. cristatum*. This phenomenon of rearrangements can also be found in the genomes of other wild relatives of wheat [62], [63], [64], [65]. Besides the genetic rearrangements of **P**-genome in *A. cristatum*, genomic rearrangements may occur in wheat–*A. cristatum* addition line due to the instability of addition line chromosome. As found in Alkhimova et al. [68] and Szakács et al. [69], the variability of wheat–rye addition line led to many rearrangements of rye chromosomes. Bento et al. [70] and Tomás et al. [71] discovered that genomic rearrangements occurred during the wheat–rye addition lines creation and appeared in the form of elimination of DNA sequences. These studies seem to suggest that some genetic differences of 6**P** chromosomes in this paper may result from elimination of DNA sequences. Sequence elimination could be reflected by the disappearance of many *A. cristatum* specific bands from some 6P addition lines. On this account, there were minor differences among 4844-12, 5113, and 5114, which belong to 6**P_I_**. In addition, we found that elimination of repetitive sequences on some chromosomes of **ABD** genomes was present in 4844-12 (unpublished results), and this situation could also be found in wheat–rye monosomic addition lines [72]. Elimination of DNA sequences might be the common form of rearrangements in addition line. Recombination between wheat genome and **P** genome did not occur in the six addition lines from the results of meiotic GISH, but we could not rule out the possibility of recombination in F_1_ hybrids. Furthermore, mobilization of active transposons induced by wide hybridization may be present, a phenomenon not examined in the present study. All in all, the genetic differences of the six wheat–*A. cristatum* addition lines might be a result of many aspects.

The genetic constitutions of different types of addition lines provide important guidance for transferring desirable compensating genes into wheat through wide hybridization. Transferring alien desirable genes and improving utilization efficiency in wheat improvement are formidable tasks due to linkage drag or insufficient compensation for the loss of wheat chromatin. To overcome this difficulty, it is necessary to identify the alien chromosomal regions of target genes and to analyze their homoeologous relationships with wheat chromosomes. Only well-compensating translocations or introgressions produced by recombination between alien chromosomal regions and homoeologous wheat chromosomes are beneficial for wheat improvement. Indeed, the 15 useful alien genes (*Lr24/Sr24*, *Sr26*, *Sr36/Pm6*, *Lr26/Sr31/Yr9/Pm8*, *Gb2/Pm17*, and *Yr17/Lr37/Sr38/Cre5*) that significantly contribute to agriculture are, without exception, compensating translocations [4], [5]. In our study, chromosome 6**P** contained many fragments that did not belong to homoeologous groups 6. If producing compensating translocations, different wheat homoeologous groups may be involved. We found that the marker *Agc26852* was specific to 6**P_I_** (4844-12/5114) by analyzing the comparative maps. Marker *Agc26852* belonged to homoeologous group 1 and mapped to bin 6**PS**-0.79–0.99; therefore, we inferred that this marker might be associated with the chromosomal region conferring high numbers of kernels per spike or powdery mildew resistance and this region also might belong to homoeologous group 1. Previous QTL mapping results have shown that a major QTL controlling high numbers of kernels per spike was located on 1**AS** of Pubing 3228, which was derived from 4844-12 [52]. The results not only suggested that genes conferring high numbers of kernels per spike had been transferred into common wheat but also were consistent with our prediction. Thus, understanding the genetic constitutions of different types of addition lines may be helpful for producing compensating translocations for gene transfer from 6**P** into the wheat genome.

The efficiency and precision of conventional breeding could be increased by means of the marker-assisted selection of objective traits [49], [50]. When breeders perform wide crosses, they have no idea of the locations of the targeted genes. They usually reduce the target region via repeated backcrossing and the use of markers linked to objective traits to accelerate the breeding process [51]. Different types of chromosome 6**P** possess different desirable genes due to rearrangements. We deduced that the marker *Agc26852* specific to 6**P_I_** (4844-12/5114) was related to the chromosomal region containing genes conferring high numbers of kernels per spike and powdery mildew resistance and mapped to bin 6**PS**-0.79–0.99. To physically locate these genes, numerous translocation lines were obtained by inducing 4844-12 using ^60^Co-γirradiation and gametocidal chromosomes [37], [53]. The marker *Agc26852* may be useful in selecting translocations with the characteristic of high numbers of kernels per spike. A large number of studies have shown that further improving wheat yield mainly depends on increasing the grain number per spike and an increasing number of specialists focus on transferring genes related to yield from wild relatives to wheat [54], [55]. Addition line II-29-2i may possess high photosynthetic efficiency because of its especially slender flag leaf and no decreasing in grain number per spike or thousand-kernel weight compared to the wheat parent, Fukuhokomugi. The improvement in photosynthesis per unit leaf area was correlated with increases in the harvest index and kernel number per square meter [56]. When other constraint conditions are not restricted, enhancing photosynthesis improves crop yield [57]. There were 187 markers specific to 6**P_III_** (II-29-2i), and 27 markers matched to mapped wheat ESTs. The latter 27 markers were distributed across different chromosomal bins and belonged to different homoeologous groups, making it difficult to determine the location and homoeologous group of the gene conferring high photosynthetic efficiency. This difficulty may be related to the rearrangements of 6**P_III_** being more complex than those of the other three chromosome 6**P** types. However, these markers may contribute to producing compensating translocations. In addition, structural rearrangements led to different phenotypes in the six addition lines. There were significant phenotypic differences between 6**P_I_** (4844-12/5113/5114) and the other three types, which is consistent with the results related to their genetic constitutions. However, there were no differences among 6**P_II_** (5106), 6**P_III_** (II-29-2i), and 6**P_IV_** (II-26) types, especially in spikelet number per spike, grain number per spikelet and grain number per spike. The reason why the phenotypes were different from the genetic constitution results may be that the differences in genetic constitution did not reflect the traits we focused on. Therefore, a greater understanding of the genetic constitutions of the six addition lines would contribute to the ability to transfer useful genes from 6**P_I_** and 6**P_III_** into the wheat genome and improve the utilization efficiency of these genes in germplasm enhancement programs.

The development of comparative maps of chromosome 6**P** will promote the development of conventional chromosome engineering toward molecular chromosome engineering. Comparative genomics has shown that gene content and order are highly conserved between related plant species [58], [59], [60]. The conservation of the content and order of genes among different species has improved the effectiveness and predictive value of information transfer. In this study, we utilized the conserved synteny between common wheat and *A. cristatum* to identify six addition lines as wheat–*A. cristatum* 6**P** disomic addition lines and to develop six comparative maps of chromosome 6**P**. The locations of STS markers on chromosome 6**P** were determined based on the locations of mapped wheat ESTs. However, the reliability of these maps should be verified using a deletion bin map of chromosome 6**P**. After all, we could not rule out the possibility of non-homologous translocation, duplication or intrachromosomal inversion of **P** chromosomes like other genome chromosomes of Triticeae [64], [65], [73]. The locations of STS markers may be not consistent with their actual physical locations. Molecular markers clustered together can effectively detect the chromosomal fragment that contains a desirable gene. Niu et al. [61] efficiently eliminated a large amount of *Aegilops speltoides* chromatin surrounding *Sr39* using DNA marker-assisted chromosome engineering. In our lab, we have produced many translocation lines of chromosome 6**P**, and these maps were used to determine the size of alien chromatin and which bin they were from (unpublished data). A similar study was conducted in the identification of 4**VS** translocations [74]. The development of comparative maps of chromosome 6**P** in this study may facilitate the transfer and elimination of alien chromatin (6**P**) tagged by molecular markers, allowing researchers to overcome the inefficiency and randomness of conventional chromosome engineering.
